# Seaweed in the Diet as a Source of Bioactive Metabolites and a Potential Natural Immunity Booster: A Comprehensive Review

**DOI:** 10.3390/ph18030367

**Published:** 2025-03-04

**Authors:** Amiya Kumar Mandal, Sudhamayee Parida, Akshaya Kumar Behera, Siba Prasad Adhikary, Andrey A. Lukatkin, Alexander S. Lukatkin, Mrutyunjay Jena

**Affiliations:** 1 Algal Biotechnology and Molecular Systematic Laboratory, Post Graduate Department of Botany, Berhampur University, Bhanja Bihar, Berhampur 760007, Odisha, India; amiyakumar0001@gmail.com (A.K.M.); sudhamayee197@gmail.com (S.P.); akshayakumarbehera54@gmail.com (A.K.B.); 2 Department of Biotechnology, Institute of Science, Visva-Bharati, Santiniketan 731235, West Bengal, India; adhikarysp@gmail.com; 3 Department of Cytology, Histology and Embryology with Courses in Medical Biology and Molecular Cell Biology, N.P. Ogarev Mordovia State University, Bolshevistskaja Str., 68, Saransk 430005, Russia; ussr1960@yandex.ru; 4 Independent Researcher, Serova Str., 3, Saransk 430004, Russia

**Keywords:** seaweed, immunity booster, bioactive compounds, anticancer, antioxidant

## Abstract

Seaweed plays an essential role in the survival of marine life, provides habitats and helps in nutrient recycling. It is rich in valuable nutritious compounds such as pigments, proteins, polysaccharides, minerals, vitamins, omega-rich oils, secondary metabolites, fibers and sterols. Pigments like fucoxanthin and astaxanthin and polysaccharides like laminarin, fucoidan, galactan and ulvan possess immune-modulatory and immune-enhancing properties. Moreover, they show antioxidative, antidiabetic, anticancer, anti-inflammatory, antiproliferative, anti-obesity, antimicrobial, anticoagulation and anti-aging properties and can prevent diseases such as Alzheimer’s and Parkinson’s and cardiovascular diseases. Though seaweed is frequently consumed by Eastern Asian countries like China, Japan, and Korea and has gained the attention of Western countries in recent years due to its nutritional properties, its consumption on a global scale is very limited because of a lack of awareness. Thus, to incorporate seaweed into the global diet and to make it familiar as a functional food, issues such as large-scale cultivation, processing, consumer acceptance and the development of seaweed-based food products need to be addressed. This review is intended to give a brief overview of the present status of seaweed, its nutritional value and its bioactive metabolites as functional foods for human health and diseases owing to its immunity-boosting potential. Further, seaweed as a source of sustainable food and its prospects along with its issues are discussed in this review.

## 1. Introduction

The marine environment occupies approximately 70% of the Earth’s surface, where more than one million multicellular organisms, from zooplankton to whales, and one billion unicellular organisms are dwelling together [1]. This not only serves as a habitat for marine organisms but is also an exceptional source of plentiful marine foods, which include seaweed and fish [2]. In addition to this, aquaculture investigators are very interested in discovering natural products with various applications, since the marine environment is a treasure trove of valuable resources. Macroalgae, or seaweeds, are multicellular algae that are visible to the unaided eye and are predominantly found in marine environments. Unlike higher plants, seaweeds are thalloid with specific structural features such as holdfasts and stipes, which operate similarly to roots and stems in certain seaweeds [3]. They can be found in all shoreline locations of the world, from the polar regions to the tropics [4]. Seaweed is classified into three categories: Rhodophyta (red algae), Phaeophyta (brown algae) and Chlorophyta (green algae) [5].

Every year, approximately 36 million tons of seaweed is harvested in nearly 40 countries. Though around 95% of the seaweed comes from Korea, China, Japan, Norway, Chile, Philippines, America, Indonesia, and India, almost 83% of the harvested seaweed is used by human beings [6]. Two million tons of seaweed was produced in the year 1969, and eventually, this increased to thirty-five million tons in the year 2019 [7]. Globally, seaweed industries use 30 to 40 million tons of seaweed annually [8]. Asia contributed 97.4 percent of the world’s seaweed production in the year 2019. Respectively, Europe and America contributed 0.8 percent and 1.4 percent in the year 2019. Globally, around 221 species of seaweed are used for a variety of purposes. Still, only 27 different seaweed species were cultivated in 2019, as per the data provided by the Aquatic and Fisheries Information System (ASFIS) [8]. In one study, it was confirmed that in India, seaweed comprises 65 families, 234 genera and 865 species. The global scenario of seaweed production, as per the Food and Agriculture Organization’s The State of World Fisheries and Aquaculture (FAO-SOFIA), 2021, is depicted in Table 1. The Indian shoreline is about 7500 km long and has large sources of seaweed. There are several zones nearby and good habitats on the Indian coast with flourishing seaweed growing with high species variety, particularly in Maharashtra, Gujarat, Tamil Nadu, Odisha, Goa, Kerala, Andaman, and Nicobar and the Lakshadweep Islands.

The unique features of marine aquaculture mean that seaweed is effortlessly grown, and they are mainly responsible for its valuable properties. Several seaweeds are edible because they do not have intrinsic toxins and contain imperative nutrients such as vitamins, fatty acids, minerals and proteins, which are essential for the growth and development of human beings [9]. With current advances in bioengineering and algal technology, various natural products have been derived from marine algae, such as proteins, terpenoids, polyamines, biopolymers, amino acids, carotenoids, phlorotannin, polyamines, fatty acids, essential minerals and growth hormones like auxin, gibberellin, cytokinin, etc. [10]. These are generally good sources of micronutrients, bioactive compounds and fibers [11] and are treated as low-calorie healthy foods [12]. Seaweed species like *Porphyra tenera* and *Palmaria palmata* are well known for their higher protein content [11]. Some published research shows that seaweed consumption has several health benefits, e.g., reducing the risks of diseases like diabetes and obesity and improving gut health [13,14,15,16,17]. Moreover, seaweed is used in food supplements (fucoidan, phlorotannin, fucoxanthin, and iodine) and food additives (FAO, 2018) to enhance the quality of food and provide health benefits.

According to the United Nations Food and Agriculture Organization (FAO), around 2 billion people are suffering from micronutrient deficiency, and approximately 842 million people suffer from hunger worldwide [18]. In this context, seaweed can meet the global deficiency. As we know, in modern society, fast food plays a vital role in our present-day life. However, fast food contains high calories with high levels of sugar, trans fats, polyunsaturated fats, white flour, several types of food additives, thickeners, preservatives, flavors and emulsifiers. Consequently, these foods are deficient in fibers, proteins and vitamins [19]. According to numerous studies, feasting on fast food also damages economic and social life. Moreover, it leads to several health problems and causes serious diseases like heart disease and obesity [20]. Numerous studies show that seaweed contains very high levels of nutrients and minerals including sodium, as well as other valuable minerals [21,22].

Seaweeds are now being used in several industries as a raw material for valuable products such as carrageenan, agar, galactans, ulvans, algin and fucoidans [23]. However, they are widely consumed directly by several nations as food. Biotic and abiotic factors such as salinity, time of collection, light intensity, herbivory intensity, life stage, phylogenetic diversity, nutrient availability, reproductive stage and species type influence seaweed diversity and metabolite content [24,25]. Seaweeds are high in minerals (iron, potassium, calcium, sodium, manganese, chromium, arsenic and selenium), vitamins (vitamins A, B, C and E), bioactive metabolites, polyunsaturated fatty acids and amino acids, but particularly they are low in calorie content [26,27]. Thus, incorporating seaweed in daily supplements can reduce the chance of several diseases, for instance, cardiovascular diseases, cancer and diabetes, along with lowering the risk of digestive health issues [28,29]. Moreover, it can be used as a useful ingredient to enhance food’s nutritious value [30]. In addition to this, seaweed has a high protein content and health benefits, which increases the interest in foods derived from seaweed [31]. Fascinatingly, researchers are revealing the effect of potential bioactive metabolites, and the phenolic molecules of seaweed are predominantly among the secondary metabolites [32]. In this review, we aimed to focus on seaweed bioactive compounds and their role in functional foods to boost immunity, as well as their biological potential as anti-inflammatory, antidiabetic, anticancer, antioxidant and antimicrobial active compounds.

## 2. Seaweed in the Food Chain

Seaweed plays an important role in the food chain and forms a promising ecosystem for the existence of many organisms in the marine ecosystem. Ocean-grown seaweed usually absorbs a variety of minerals and many other nutrients. It impartially produces simple foods and is easy to digest by human beings, along with releasing beneficial substances that provide diverse types of minerals and vitamins [33]. Seaweed produces slime excretions, which are very rich in polysaccharides, sugars, and amino acids, that are consumed by micro-organisms like bacteria and also by marine organisms such as sharks, sponges, snails, bivalves, sea urchins and fish like tilapia and carp that directly eat the seaweed or consume it by the filter-feeding process [9]. Eventually, the food is passed to different trophic levels and reaches human consumption. Records of using seaweed as feed for cattle in the Mediterranean basin also provide valuable information about seaweed [9]. *Macrocystis pyrifera* and *Gracilaria edulis* (Rhodophyta) are used for abalone feed in Australia [34]. Several articles have mentioned that seaweed protein extract is also being used as fish feed. Seaweed like *Grateloupia filicina*, *Porphyra yezoensis*, *Gracilaria* sp., *Ecklonia cava*, *Laminaria japonica*, *Undaria pinnatifida* and *Porphyra tenera* are traditionally consumed as sea vegetables [35]. Moreover, seaweed extracts of *Sargassum* sp., *Halimeda microloba* and *Turbinaria ornate* are also being used as fertilizers for plants, as well as growth stimulators. Some studies suggest that liquid seaweed extracts at low concentrations work better than chemical fertilizers [36].

## 3. Nutritional Food Value of Seaweed

**Figure 1:**
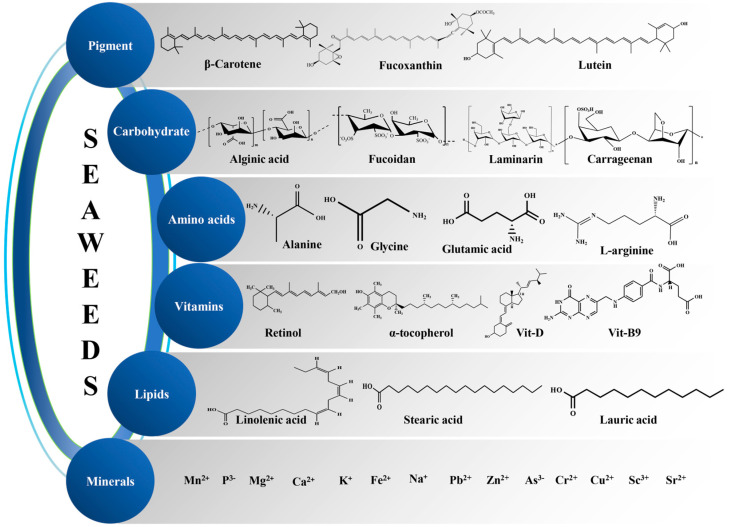
Seaweed-based bioactive compounds.

Bioactive compounds that originate from seaweed have captivated the interest of scientists due to being natural, allowing them serve as a substitute for artificial substances [37]. Several studies confirm that seaweed is a good source of nutritious food. It contains fewer calories and is very rich in health-promoting high-value products such as pigments, carotenoids, vitamins, proteins, polysaccharides, essential amino acids, omega-3 rich oils, phenolics, flavonoids, polyphenols and sterols, which prevent the onset of cancer, diabetes, inflammatory diseases, Alzheimer’s, Parkinson’s and cardiovascular diseases [33]. Seaweed-based bioactive compounds are represented in Figure 1. Therefore, seaweed has great potential to be used in nutraceuticals. Different bioactive compounds obtained from seaweed are summarized below.

### 3.1. Bioactive Compounds from Seaweed

#### 3.1.1. Pigments

The pigments present in seaweed are chiefly categorized into different types, such as carotenoids, phycobiliproteins and chlorophylls. The seaweed carotenoids include fucoxanthin, carotenes, zeaxanthin, lycopene, neoxanthin, lutein and astaxanthin [38], whereas seaweed phycobiliproteins mainly include phycoerythrin (red pigment), phycocyanin (blue pigment) and allophycocyanin (light blue pigment) [38]. Moreover, seaweed chlorophylls are fat-soluble green pigments that play a crucial role in photosynthesis [38]. These pigments are a potential source of food colors, nutraceutical ingredients, and biologically active agents with immunomodulatory, antidiabetic, anticancer, antioxidant, anti-inflammatory and antiangiogenic properties [38]. Seaweed pigments with benefits belonging to different groups of seaweed are presented in Table 2.

#### 3.1.2. Proteins

Depending upon the species, the protein content of seaweed varies considerably. A higher percentage of protein is found in red seaweed, followed by green and brown seaweed, which constitutes around 47%, 9 ± 26% and 3 ± 15%, respectively [79]. For example, *Porphyra yezoensis* and *Palmaria palmata* contain protein at up to 47% and 35% of the dry weight, which is found to be more as compared to higher plants like *Glycine max* in some cases [80]. However, seasonal variations and environmental factors such as temperature and light play significant roles in seaweed protein content [25]. A study by Fattah and Sary (1987) showed that *Ulva lactuca* contains more protein in August than in April, which supports the findings of Augier and Santimone (1978) [81,82]. The protein content of red seaweed found in the northern hemisphere increases in winter and spring than in summer due to high nitrogen levels [83]. Apart from this, *Kappaphycus alvarezii* originating from India and Malaysia was found to have 18.2% and 8.8% proteins, respectively, which implies that geographical location also influences the protein content [84,85].

The amino acid profiles of different seaweeds confirmed the presence of amino acids such as arginine, lysine, leucine, aspartic acid, glutamic acid and threonine [86]. Aspartic acid and glutamic acid are the major amino acids found in brown seaweed and green seaweed [87]. Around 26–32% of the total amino acids comprises aspartic acid and glutamic acid in *Ulva* sp. [88]. Among brown seaweeds, *Fucus* sp., *Laminaria digitata* and *Ascophyllum nodosum* contain high levels of these amino acids [89]. In contrast, *Undaria pinnatifida* contains high levels of methionine and arginine in addition to other amino acids such as glycine, alanine, aspartic acid and glutamic acid [90]. Sulphur-containing amino acids have a vital role in protein synthesis. Red seaweeds such as *Porphyra acanthophora*, *porphyra tenera*, *Chondrus crispus* and *Grateloupia turuturu* are rich in essential amino acids, methionine and cysteine [91,92].

Carnosine, a peptide found in *Acanthophora delilei*, is generally present in animal muscle and possesses antioxidant activity, which can be a modulator of diabetic and Alzheimer’s disease [93]. Taurine is another rare amino acid found in *Gracilaria textorii*, and *Gracilaria vermiculophylla* boosts physical energy and enhances mental performance along with preventing the absorption of heavy metals in the stomach [84]. *Palmaria palmata*, *Undaria pinnatifida*, *Porphyra yezoensis* and *Porphyra columbina* are some seaweeds possessing antihypertensive properties by inhibiting angiotensin-converting enzyme which generally regulates blood pressure [94]. In addition to this, anti-inflammatory activity is displayed by the hydrolysates of different seaweeds, which is associated with the upregulation of interleukin 10 and inhibition of TNFα (tumor necrosis factor-alpha) expression in *Ulva* sp. and the inhibition of NF kB expression in *Pyropia columbina* [95,96]. In Japan, the claim for antihypertensive activity has already been considered, and the application of red seaweed as the source of peptides has been approved by Foods for Specified Health Uses (FOSHU) [84]. However, the use of purified protein in food industries from seaweed is limited. Proteins from red seaweed, such as R-phycoerythrin and R-phycocyanin, have higher digestibility than other groups due to the presence of low antinutrients [97]. But then, the digestibility of seaweed proteins is generally moderate due to the presence of carbohydrates and antinutrients, hence requiring pretreatment including freezing, drying, ensiling and specific extraction procedures, including enzymatic extraction [98]. The protein contents in different seaweeds are illustrated in Table 3.

#### 3.1.3. Carbohydrates

Seaweed is considered a good source of carbohydrates and is used in industries that are obtained from cultivated sources or natural environments [113]. Carbohydrates from seaweed mainly consist of polysaccharides, a few monosaccharides and disaccharides [114] depending upon the species, time, harvesting site, age of the species, etc. Red seaweed is rich in carrageenan, xylan, agar and galactan. Brown seaweed is rich in alginate, fucoidan and laminarin, whereas green seaweed is a rich source of ulvans [59,115,116,117]. There are mainly two types of polysaccharides found in seaweed in the storage and structural forms. The structural polysaccharides of seaweed are quite similar to those of terrestrial plants and primarily include hemicellulose, cellulose and xylan; however, alginate, carrageenan, laminarin, fucan, ulvan and agar come under the storage form of polysaccharides. Seaweed-derived polysaccharides are used as thickeners, stabilizers and emulsifiers in the food business because of their gelling properties. Some foods’ texture and shelf life may be enhanced by them [118]. These are mainly used in coffee, toothpaste, shampoo and frozen poultry. The biomedical application of different polysaccharides extracted from various species is presented in Table 4.

- Structural polysaccharides

The health benefits of cellulose and hemicellulose have already been confirmed by numerous studies. These polysaccharides help in reducing the risk of heart disease and cancer and are anti-inflammatory. Moreover, they also improve the immune system and gut health by fermenting metabolites. The cell wall component, xylan, because of its biocompatibility, can be used in a variety of biomedical applications, such as wound healing, tissue engineering and pharmaceutical formulations for drug delivery systems because of their ability to form gels and encapsulate active components [206,207]. Additionally, it is used in biotechnology as a substrate for the synthesis of biofuels since it can be converted to sugars by microbes and then fermented to produce bioenergy [208,209].

- Storage polysaccharides

There are three substantial forms of carrageenan, mainly lambda, kappa and iota. In addition to having different health benefits, it is used in tissue culture medium as gels [210]. Agar is especially well liked as a gelatin alternative in vegetarian or vegan dishes [211]. Desserts, jellies and some confectionaries are popular places to find it. Agar may have certain health advantages, but it is most recognized for its use in science and cooking [212]. As a solidifying agent, it is used in laboratory and plant tissue culture medium [213,214,215]. Eventually, agar is utilized in biotechnological processes including the synthesis of medicine capsules and gel electrophoresis [216,217]. Because of its capacity to create a gel that aids in moisture retention, it can also be utilized in wound dressings [218,219].

Galactans, ulvans and alginates can be used in pharmaceutical formulations for drug delivery methods. Because of their gelling and viscosity-enhancing qualities, they aid in the controlled release of medications [220]. These are useful in biotechnology, especially in the fields of tissue engineering and regenerative medicine [206,207]. They can be included in cell culture scaffolds. Ulvans improve the stability and texture of goods, including dairy products, sauces and dressings [221]. It is important to remember that even while ulvans may have health benefits, research on them is still in its early stages, and further studies are required to understand their mechanisms and efficacy completely. The food and pharmaceutical sectors have taken an interest in fucoidan, a marine acid polysaccharide, because of its possible medical applications. Fucoidan is a polysaccharide that mostly consists of L-fucose and sulfate groups. Its distinct biological structure is responsible for its exceptional biological activity. Like β-glucan, laminarin is a strong antioxidant that acts via several different mechanisms and controls cell division and death [222]. It also demonstrates a possible therapeutic impact on colon cancer in humans [223,224]. The wound-healing properties of laminarin are associated with antimicrobial activities and macrophage stimulation.

The nanoporous nature of alginates makes the drugs diffuse quickly through them and release small regeneration agents through diffusion because their tiny pore sizes are chemically modifiable with little effort [225]. As alginate gels do not include mammalian cell receptors and have low protein adsorption, they are being used more and more in biomedical research as model systems for mammalian cell culture [226,227]. The release of heparin-binding growth factors such as vascular endothelial growth factor (VEGF) has been utilized to induce blood vessel development [228]. Alginate gels have shown promise in tissue engineering and regeneration, including in the liver, pancreas, skeletal muscle and nerve [229]. They have also been used in cell-based neurological therapeutics, tissue engineering and nervous system repair [230]. Moreover, they have also been used in the treatment of Type I diabetes [231].

#### 3.1.4. Lipids

Lipids are fatty acids essential for humans and are composed of saturated, monounsaturated and polyunsaturated fats [232]. The lipid profile of seaweed contains a wide number of polyunsaturated fatty acids (PUFAs), such as linoleic, lauric acids, linolenic acids, stearic acids and docosahexaenoic (DHA), with well-recognized health benefits [233,234,235]. Marine seaweed is the main nutritional constituent of sterols. Sterols are chiefly represented by clionasterol, cholesterol, fucosterol and isofucosterol [235,236,237]. Depending on many factors, such as season, salinity, geographical area, temperature, light, climatic conditions and seaweed species, seaweed lipid content and fatty acid composition can change significantly [25]. However, seaweeds have complex lipids, including phospholipids and glycolipids that are esterified with omega-3 fatty acids like docosahexaenoic acid and eicosapentaenoic acid, unlike terrestrial plants [238]. Complex lipids have recently come into focus as prospective phytochemicals with underlying bioactive qualities, such as antibacterial, anticancer, anti-inflammatory and antioxidant, which might lead to potential uses in the pharmaceutical, nutraceutical and cosmeceutical industries [238].

Increasingly, essential fatty acids are being recognized as functional foods and nutraceuticals with many health benefits, as well as reducing the risk of cancer, cardiovascular diseases, osteoporosis and diabetes [239]. In addition, coronary heart disease is directly linked to the development of atherosclerosis, which narrows coronary arteries and is triggered by connections between lipoproteins, endothelium, monocytes, plasma lipids, platelets and smooth muscle of arterial walls [240]. According to several reports, the consumption of lipids in Western countries is relatively high, which contributes about 40% of the total calories required for individuals [241]. However, in general, seaweed is a good source of healthy lipids. Omega-3 PUFAs are more abundant than omega-6 PUFAs in the majority of brown seaweed lipids [242]. Due to this high percentage of omega-3 PUFAs, brown seaweeds might be used to alter the omega-6/omega-3 ratio of dietary lipids [243]. The biological applications of seaweed lipids are represented in Table 5.

#### 3.1.5. Vitamins

The vitamin status of feed and diets is improved by seaweed, which is a significant source of water-soluble and fat-soluble vitamins. Moreover, it contains water-soluble vitamins like pantothenic acid, niacin, riboflavin, folic acid and vitamin C, in addition to fat-soluble vitamins like vitamin A, vitamin D and vitamin E [258,259,260]. *Porphyra umbilicalis* and *Gracilaria changii* are good sources of vitamins B_12_ and B_3_, respectively [261,262]. *Sargassum polycystum*, *Ulva lactuca*, *Caulerpa lentillifera*, *Eucheuma cottonii* and *Gracilaria* sp. are rich in water-soluble vitamins, including vitamin C, which help to prevent the development of atherosclerosis and low-density lipoprotein oxidation [263]. Studies report that the red algae *Gracilaria chilensis*, *Macrocystis pyrifera* and *Codium fragile* have significantly higher levels of dried carotene (e.g., 113.7 mg/g, 17.4 mg/g and 197.9 mg/g), respectively, than other vegetables [264]. But compared to green and red seaweed, the brown seaweed *Undaria pinnatifida* has higher levels of vitamin E [11]. Compared to terrestrial plants, seaweeds like *Crassiphycus changii*, *Porphyra umbilicalis* and *Himanthalia elongata* are rich in vitamins [29]. Interestingly, *Ascophyllum* sp. and *Fucus* sp., brown seaweeds, have higher levels of vitamin E than other green and red seaweeds [265].

Numerous biological processes involving vitamins include coenzymes, antioxidants, hormones, cell signaling regulators and their regulation of tissue and cell proliferation [266]. Vitamin A or carotene is used in cosmetic industries to reduce hyperpigmentation on the face, as well as having antioxidant and anti-wrinkle properties [267]. Higher amounts of the vitamin B complex (B1, B2, B3, B6, B9 and B12) are present in red or green seaweed [268]. The anti-aging properties of vitamin B12, which is required for hair and nail growth, were previously confirmed in red seaweed [269]. Moreover, ascorbic acid plays a vital role in algae’s photoprotection and primarily in the photoprotective xanthophyll cycle (violaxanthin, diatoxanthin, antheraxanthin and zeaxanthin) [270]. Ascorbic acid regulates the quantity of hydrogen peroxide produced within the cell during photosynthesis by removing the hydrogen peroxide produced by the photo-absorption of oxygen in PSI [271]. This vitamin possesses antioxidant, antiviral, anti-inflammatory, antibacterial, detoxifying and anti-stress properties when applied topically. It might be used to enhance tissue growth and the development of teeth and bones and repair blood vessels [272]. In addition to having several health benefits, it is used in the cosmeceutical industry for functional food [273]. Several studies have reported a high vitamin C content in the red algae *Porphyry leucosticta* and *Ceramium rubrum*. Moreover, vitamin E reduces the risk of cardiovascular disease and prevents the oxidation of low-density lipoprotein [272].

#### 3.1.6. Minerals

A sufficient mineral intake on a regular basis is crucial for preventing degenerative and chronic nutrition-related illnesses. Moreover, a high percentage of minerals are found in seaweeds, which are accumulated from seawater depending on seasonal variations and environmental conditions. Phaeophyta has a higher rate of absorption as compared to Rhodophyta and Chlorophyta due to the presence of alginic salts, alginic acid and alginate [274]. Consequently, the mineral content of seaweeds is at least 10 times that of terrestrial plants and exceeds 20–50% by dry weight [115,275,276]. Seaweed has a substantial amount of micro (Pb^2+^, Zn^2+^, As^3−^, Cr^2+^, Cu^2+^, Sc^3+^ and Sr^2+^) and macro (K^+^, Ca^2+^, Mn^2+^, Mg^2+^, Fe^2+^, P^3−^ and Na^+^) elements [115,117]. Therefore, seaweeds can contribute greatly to the daily intake of minerals and are a promising source of functional foods, nutraceuticals and food supplements. There is even a possibility that they might help to solve the global mineral deficiency among humans (for instance, Fe, Zn and I). It is estimated that a gram of dry seaweed has a higher level of these minerals than other sources of mineral-rich foods and can supply a significant portion of the recommended daily allowance (RDA) or adequate intake (AI) [21].

According to reports, a high Na/K ratio diet is associated with hypertension, making this a nutritionally significant issue. Hence, a low Na/K ratio, which is around 0.14–0.16, makes seaweeds a balanced Na and K source [277]. The green seaweed *Ulva clathrata* is an exception since its high salt concentration increases the Na/K ratio. In contrast, red and brown seaweeds, with the exclusion of *Undaria pinnatifida*, have lower Na/K ratios. Moreover, this seaweed also has antihypertensive properties, which is attributed to the presence of some peptides that suppress the angiotensin-1-converting enzyme, which eventually controls blood pressure [277]. Furthermore, anemia is a worldwide health concern caused by iron deficiency associated with inadequate food consumption, blood loss and malabsorption [278]. Iron-deficient people may benefit from the iron-rich red seaweed *Pyropia tenera* and the green seaweed *Codium fragile* [279].

Another worldwide concern is iodine deficiency which raises the risk of thyroid malfunction, hypothyroidism, mental retardation, diminished cognitive function and productivity at work. Iodine plays a major role in the production of thyroid hormones like tri-iodothyronine and thyroxin, which are necessary for the body’s growth, nutrition utilization and organ development [280]. According to estimates from the WHO, 1.6 billion people are in danger of iodine shortage, and at least 20 million of them have mental abnormalities that can be avoided by treating iodine deficiency. Brown seaweeds are known for having a high iodine content, and *Laminaria* sp. is a substantial source of iodine. Among others, the green seaweed *Ulva clathrata* and the red seaweed *Gracilaria* sp. have a high iodine content. In addition, the functioning of over 300 enzymes and 1000 transcription factors depends on zinc, an important micronutrient that is also crucial for the host’s defense against infections [281,282]. Zinc concentration is high in seaweeds, especially red and brown seaweeds. The amount of zinc can be as high as 0.70 mg/g, depending on the region and species type. Hence, seaweed could potentially be used in the production of functional foods due to its high level of important minerals.

## 4. Seaweed as an Immune Booster

**Figure 2:**
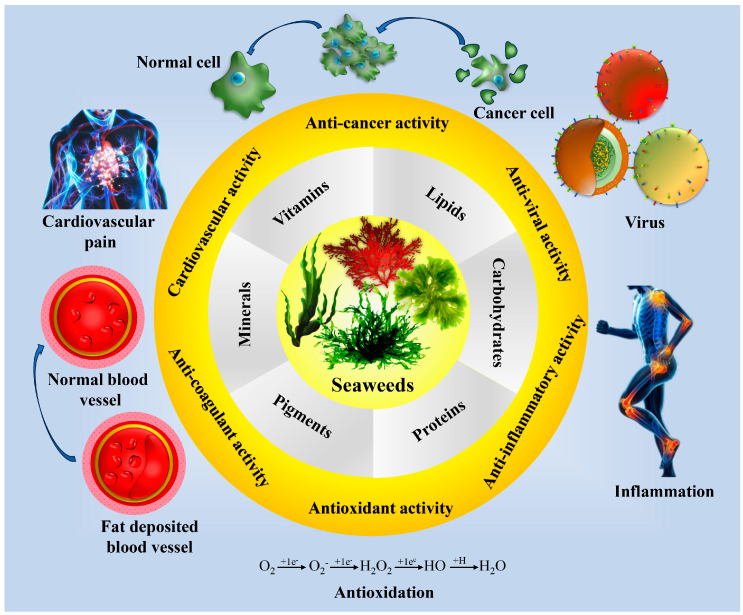
Biological properties of seaweed.

Seaweed’s chemical composition is similar to that of human plasma and plays an excellent role in the purification of blood and regulating it [283]. Eventually, it helps to alkalinize human blood and neutralize the over-acidic effect. Several studies confirm that chlorophyll-rich seaweed is more influential as a natural detoxifier, usually facilitating the removal of waste products from the bloodstream [284]. Seaweed-derived bioactive compounds have a decent activity promoting good health for several years, and they seem to be a good bioactive nutrient component, as depicted in Figure 2. They can alter the genetic appearance of the host by prompting cellular activity, influencing good health and providing antioxidants as well as several enzymes (Viscozyme, Flavourzyme, Neutrase, Celluclast and Termamyl, etc.) for inhibitory activity [285,286].

Seaweed-based polysaccharides are unique because of their origin, having immunological properties extending to the human immune system and subsequently acting as antiviral, antitumor and anti-infective compounds. Some polysaccharides exert their action by acting as antimutagens and antioxidants. Furthermore, hematopoietic activity is shown by some seaweed polysaccharides [287,288,289]. Sodium alginate, a seaweed polysaccharide from *Undaria pinnatifida* and *Laminaria japonica*, when used for medicinal purposes as an antiviral agent, showed very little cytotoxicity to mammalian cells and acted as an antinutritional factor by reducing the action of digestive enzymes [290]. However, it showed excellent immune modulator properties allied with antitumor properties. Sulfated polysaccharides from seaweed have a role as anti-neoplastic agents. Numerous investigations have reported that seaweed and sulfated polysaccharides have the role of antiproliferative properties in tumor inhibition, as well as in cancer cell lines [291]. Polysaccharides extracted from *Sargassum stenophyllum*, a brown marine alga, have inhibitory effects on developmental angiogenesis and vasculogenic processes in chick embryos [292]. Polysaccharides extracted from *Gracilaria verrucosa* and *Porphyra yezoensis* have an impact on phagocytosis stimulation and macrophage respiratory burst in mice under laboratory conditions [293,294]. These polysaccharides also have promising anti-inflammatory activities [295]. Macrophage stimulation by seaweed-extracted polysaccharides plays a crucial role in immune stimulation. Macrophages are present in immune cells in the inborn immune system, and the stimulation of macrophages plays a pivotal role in the maintenance of homeostasis by changing their activity based on the tissue. Marine red algae extract produces carrageenan, which has a potentially inflammatory effect of producing tumor necrosis factor in action with lipopolysaccharides from bacteria [191]. The potential biological activity possessed by seaweed-derived bioactive compounds is presented in Table 6.

## 5. Seaweed as a Sustainable Source for Humans

Seaweed grows abundantly in the seas and oceans in their natural environment. However, it does not need any fertilizers or artificial nutrient media to grow, therefore keeping its ecological impact negligible [2]. Seaweed has a high productive and reproductive rate when precise techniques are used. In addition to this, it does not need any agricultural land for its cultivation. Several studies confirm that more than 10,000 species of marine algae exist in all climatic conditions, including polar ice or warm tropics regions. The cultivation of seaweed for food has been continued for several centuries in China, India, Japan, South Korea, Tanzania, North Korea, Vietnam, etc.

In many countries, coastal communities have a tradition of eating seaweed. Mostly in Eastern Asia, seaweed is frequently and widely consumed in salads, snacks, soup ingredients, sushi wraps, etc. Many seaweeds are very tasty when properly prepared, and they are integrated into dietary habits in modern cuisine in many forms, such as dried, raw or cooked. However, they are also consumed in nutrient- and flavor-packed food [348]. The prospects of the use of seaweed as food can be found in Japan in the 4th century and in China in the 6th century. Presently, China, Japan, and Korea are the major consumers of seaweed in the world. In addition to this, seaweed is also used as a biofertilizer in horticulture and agriculture, food for aquaculture, fodder for animals, etc. Seaweed, like *Palmaria* sp., has been used as a food for the coastal populations of Ireland; Dulse was used as a condiment with bread, butter and milk [349]. Eventually, all of these aspects make seaweed an outstanding sustainable source of food. On the other hand, the seaweed consumption in India along the coast of Tamil Nadu and Kerala is relatively less.

Approaches are required to introduce seaweed into Indian cuisine and make it attractive not only based on a health point of view but also based on tastiness to the people for the fulfillment of nutrients and essential minerals requirements. A critical valuation and survey are required for the aroma and different flavors of raw, processed and dried seaweeds concerning their taste. However, it is essential to recognize the science behind the development and enhancement of flavor in seaweed to detect components for flavor and taste. In addition to this, cooking and processing techniques also need to be focused on. Most importantly, the consumers of India should accept and use healthy multipurpose seaweed compounds and beneficial products from seaweed with their present trends of lifestyle.

## 6. The Biotechnological Advancements in Seaweed-Based Food Supplements

Commonly, cereal-based products are consumed directly due to their low cost, easy preparation and long usefulness for consumption as well. Though meat is a valuable source of vitamins and proteins, it does not contain a sufficient amount of fiber and has extreme levels of sodium that are harmful to humans. Nevertheless, its health benefits and nutritional quality could be improved by the addition of seaweed-based bioactive substances [350]. Several studies confirm that seaweed can be used as food as well as supplying a high amount of fiber to the body. The quality of bread was enhanced by the addition of bioactive compounds derived from *Ulva* sp. and a powder of *Laminaria* sp. [351]. Lipid powder and flour derived from seaweed are widely used as main components in modern cuisine instead of eggs [352]. However, the amount of seaweed that needs to be consumed depends on its compositional information since some elements, like iodine, cause harmful effects when they are consumed in excess amounts [353]. For example, iodine, when consumed in excess, may cause hyperthyroidism, hypothyroidism and iodism, and limiting the iodine intake altered iodine-induced goiters in the Japanese population [354]. In addition to this, the presence of heavy metals and their metabolites like arsenosugars and their metabolites in urine samples after seaweed intake has also been confirmed by previous reports [355]. Naidu et al. (1993) investigated the effect of seaweed toxicity by feeding powder samples of *Sargassum johnstonii*, *Enteromorpha linza*, *Ulva fasciate* and *Caulerpa taxifolia* to rats, and lectins were found to be the reason for toxicity—resulting in growth retardation [356].

An increased antioxidant activity in low salt-containing meat samples supplied with polyphenolic compounds from wakame and nori has already been observed [357]. Softer beef patties with low cooking loss by the addition of wakame; an increased shelf life of chicken meat and its products by the addition of fucoxanthin [30]; an improved antioxidant activity of fish and fish products by the addition of phlorotannins from *F. vesiculosus* [358]; wakame pasta with high antioxidant activity; and *Sargassum marginatum* pasta with increased reducing power are certain examples of using seaweeds as a supplementary food ingredient [359]. Furthermore, the preparation of seaweed in the form of soup with a high iodine concentration; chocolate with 40–50% iron; pickle and pakoda with higher nutrition; spices with high protein, fiber and ash content; noodles with high nutrient content; wafer, porridge, jelly, jam, coffee with higher antioxidant activity; and cookies and sauce have been reported previously [280]. The addition of seaweeds such as *Enteromorpha*, *Undaria pinnatifida* (wakame) and *Porphyra umbilicalis* (nori) to meat products and cereal-based products results in explicit changes in the antioxidant activity of the final product [360,361]. In addition to these, oils in combination with seaweed extracts displayed a delay in oxidation when *Grateloupia filicina* extract in combination with linoleic acid and fish oil was administered in rat lymphocytes [254,362]. The inhibition of lipid peroxidation was also observed by Siriwardhana et al. (2004) [363]. Moreover, the antibacterial properties possessed by seaweeds due to the presence of specific compounds like polyhydroxylated fucophlorethol, 3-bromo-4,5-dihydroxybenzaldehyde, 12 S-hydroxybromospha-erodiol, bromosphaerone and bromophenols, as well as antifungal activity due to taondiol, can be beneficial for the preservation of food [364,365,366].

Furthermore, seaweed is a rich source of biopolymers like polysaccharides that can be used in biomedical and food industries as dispersant, scaffold, coating, packaging, thickening, gelling and stabilizing agents due to their biodegradability, biocompatibility and high water-holding capacity [367]. Moreover, coatings and composite films produced from seaweed are used for food packaging [368]. Though biotechnology industries based on seaweed are growing with improvements in aquaculture techniques that produce a huge amount of seaweed biomass, such as *Porphyra* sp., *Gracilaria* sp. and *Laminaria* sp., and seaweed-based phycocolloids are utilized for the development of industries such as the carrageenan, algin and agar industries, there is a large gap in using seaweeds in food industries [369,370]. In addition, their incorporation into food requires a toxicity evaluation and studies regarding their interaction with the body metabolism by in vivo studies.

## 7. Conclusions

Seaweed is extensively known for its potential bioactive compounds, which have extensive therapeutic properties and improve a balanced diet if regularly consumed. Seaweed intake also decreases the mortality rate, recent studies have suggested. Numerous studies have verified that adding bioactive components from certain seaweeds improves foods’ nutritional value. On the other hand, the excess consumption of seaweed is also associated with certain issues such as heavy metal contamination, digestive problems because of their high fiber content, allergic reactions and thyroid dysfunction due to the overconsumption of iodine-rich seaweed. However, proper investigation of the moderate quantity to be taken in the meal can help to overcome such issues. Though whole seaweed is consumed in several countries, its incorporation into diets in different regions is still unfamiliar. Moreover, further investigations are needed to use it as a fortified food, retaining its nutritional value. For integration into global diets, the large-scale cultivation, processing, distribution, consumer acceptance and development of innovative seaweed-based food products require further studies. Along with these, the contamination level needs to be minimized in farmed seaweed. By addressing these gaps, seaweed may be the most possible way to achieve the goal of developing an alternative form of sustainable food for human beings.

## Figures and Tables

**Table 1:** Global scenario of seaweed production (source: FAO-SOFIA, 2021).

Country	Seaweed Productions (2019)	
	Wet Weight (In Tons)	Total Percentage of World Production
China	20,296,592	56.75
Indonesia	9,962,900	27.86
South Korea	1,821,475	5.09
Philippines	1,500,326	4.20
North Korea	553,300	1.71
Chile	426,605	1.19
Japan	412,300	1.15
Malaysia	188,110	0.53
Norway	163,197	0.46
Tanzania	106,069	0.30
Vietnam	20,300	0.06
Russia	19,544	0.05
Solomon Islands	5600	0.02
India	5700	0.02
Madagascar	9665	0.03
Papua New Guinea	4300	0.01
Kiribati	3650	0.01
Total global production	35,762,504	

**Table 2:** Potential benefits of seaweed pigments.

Pigment	Biological Activities	Algal Source	References
β-Carotene	Antioxidant potential in vivo, antimutagenic, provitamin A	Phaeophyta, Rhodophyta, Chlorophyta	[39,40,41,42,43]
Fucoxanthin	Antioxidant food additives, antidiabetic properties, anti-obesity properties, antihypertensive activity and lower stroke risk factors, prevent osteoporosis, anticancer activities, prevent breast cancer, immune-modulatory, antiangiogenic, antimalarial activities, neuroprotective and anti-inflammatory property	Phaeophyta	[44,45,46,47,48,49,50,51]
Zeaxanthin	Antioxidant potential in vivo	Rhodophyta, Chlorophyta	[41,52,53]
Lutein	Antioxidant potential in vivo, antimutagenic, protects eyes from oxidative stress, prevents heart disease	Rhodophyta, Chlorophyta	[41,54,55]
Astaxanthin	Antioxidant, immune-modulatory, antiangiogenic, antimalarial activities, and anti-inflammatory effects, antioxidant, and immune-enhancing properties	Chlorophyta	[56,57,58,59]
Phycoerythrin	Antioxidant, anticancer activity	Rhodophyta	[60,61,62]
Phycocyanin	Anticancer activity, antidiabetic and anti-inflammatory activity, antimicrobial activity	Rhodophyta	[63,64,65]
Pheophorbide a	Antioxidant	Rhodophyta	[65,66]
Phycoerythrobilin	Antioxidant	Rhodophyta	[67,68]
Siphonaxanthin	Anticancer, antiangiogenic	Chlorophyta	[53,69]
Chlorophyll a	Antioxidant, antimutagenic	Phaeophyta, Rhodophyta, Chlorophyta	[55,70,71]
Neoxanthin	Antiproliferative activity, anticancer activity, anti-inflammatory activity, anti-obesity properties, antioxidant activity	Chlorophyta	[53,58,72,73]
Violaxanthin	Antiproliferative activity, anti-inflammatory activity, antioxidant activity	Chlorophyta	[74,75]
Antheraxanthin	Antioxidant activity, anticancer activity	Chlorophyta	[76,77,78]

**Table 3:** The concentration of proteins in seaweed.

Name of Seaweed	Protein Content (Dry Weight in %)	References
Chlorophyta		
*Caulerpa lentillifera*	9.5–20.4	[99,100]
*Cladophora glomerata*	14.1–20.4	[101,102]
*Caulerpa sertularioides*	20.0	[103]
*Enteromorpha compressa*	12.3	[102]
*Enteromorpha flexuosa*	7.9	[99]
*Enteromorpha intestinalis*	15.2–16.4	[101,104]
*Ulva fasciata*	6.6–8.8	[99,103]
*Ulva lactuca*	3.3–27.2	[104,105,106,107]
*Ulva reticulata*	13.5–20.0	[102,108]
Phaeophyta		
*Dictyota acutiloba*	12	[99]
*Dictyota sadvincensis*	6.4	[99]
*Laminaria* sp.	7.5	[109]
*Padina gymnospora*	11.2–17.1	[103,104]
*Padina pavonica*	13.6	[102]
*Sargassum echinocarpum*	10.3	[99]
*Sargassum obtusifolium*	13	[99]
*Sargassum vulgaris*	16.3	[103]
*Undarina pinnatifida*	19.8	[109]
Rhodophyta		
*Amansia multifida*	25.6	[103]
*Bryothamnion seaforthii*	17.3	[103]
*Corallina officinalis*	2.3–6.9	[103,110]
*Enantiocladia duperreyi*	19.5	[103]
*Gelidiella acerosa*	31.1	[102]
*Gracilaria birdiae*	7.1	[111]
*Gracilaria folifera*	7	[104]
*Gracilaria salicornia*	5.6	[99]
*Hypnea charoides*	18.4	[106]
*Hypnea japonica*	19.1	[106]
*Laurencia filiformis*	18.3	[111]
*Palmaria palmata*	18.3	[112]
*Porphyra* sp.	31.3–44	[109,110]
*Solieria filiformis*	21.3	[103]
*Vidalia obtusiloba*	18.1	[103]

**Table 4:** Seaweed polysaccharides and their biological properties.

Polysaccharides	Algal Sources	Properties	References
Carrageenan	*Gigartina skottsbergii*	Antiviral, inhibits influenza virus	[119]
	*Meristiella gelidium*	Antiviral	[120]
	*Acanthophora spicifera*	Antiviral against HSV-1, inhibits virus replication	[121]
	*Hypnea musciformis*	Anticancer	[122]
	*Champia feldmannii*	Antitumor	[123]
	*Stenogramme interrupta*	Antiviral	[124]
	*E. spinosa*	Anticoagulant, antithrombotic	[125]
	*Chondrus crispus*	Antiviral, anticoagulant, antithrombotic	[126]
	*C. ocellatus*	Antitumor	[127]
	*C. ocellatus*	Antitumor	[128]
	*Gigartinaceae* *Tichocarpaceae algae*	Antioxidant	[129]
	*Tribonema minus*	Anticancer	[130]
	*Padina tetrastromatic*	Anti-inflammation	[131]
	*G. skottsbergii*	Antiviral	[132]
Xylans	*Sebdenia polydactyla*	Antiviral	[133]
	*Scinaia hatei*	Antiviral	[134]
	*Caulerpa lentillifera*	Antioxidative and antitumor	[135]
	*Spatoglossum schröederi*	Antithrombotic; peripheral anti-nociceptive; antiproliferative, anti-adhesive, antioxidant	[136]
Agar	*Acanthophora spicifera*	Antiviral against HSV-2, inhibits the initial attachment of the virus to the cells	[137]
	*Gelidium amansii*	Antioxidant activity	[138]
	*Gloiopeltis complanata*	Antiviral	[139]
	*Cryptopleura ramosa*	Antiviral	[140]
	*Bostrychia montagnei*	Antiviral	[141]
	*Gracilaria corticata*	Antiviral	[142]
Galactans	*Callophyllis variegate* *Agardhiella tenera* *Schizymenia binderi* *Cryptonemia crenulata*	Antiviral	[143]
	*Laminaria japonica*	Anti-lipidemic, antiviral, antitumor, immunomodulator, antioxidant neuroprotective	[144,145]
	*Sargassum* sp.	Antitumor	[146]
	*Adenocystis utricularis*	Antiviral	[147]
	*Spatoglossum schröederi*	Antithrombotic	[148]
	*Schizymenia dubyi*	Antiviral	[149]
	*S. binderi*	Anticoagulant	[150]
	*Grateloupia indica*	Anticoagulant, antithrombotic	[151]
	*Gymnogongrus torulosus*	Antiviral	[152]
	*Gigartina acicularis*	Antioxidant	[148]
	*Euchema cottonii*	Antioxidant	
	*Aghardiella tenera*	Antiviral	[153]
	*Gelidium crinale*	Anticoagulant	[154]
	*Porphyra* sp.	Antitumor, hypotensive, regulates blood cholesterol	[155]
Ulvans	*Enteromorpha compressa*	Antiviral against HSV, inhibits the adsorption and replication of the virus	[156]
	*Ulva intestinalis*	Antiviral against measles virus, reduction in syncytia formation and low cytotoxicity	[157]
	*U. armoricana*	Antiviral against HSV-1	[158]
	*U. pertusa*	Antioxidant, antiproliferative, hypocholesterolemic	[159]
	*U. rigida*	Immunostimulatory	[160]
	*U. pertusa*	Antioxidant and antihyperlipidemic activity	[130]
	*U. pertusa*	Antioxidant activity	[161]
	*Ulva* sp.	Anti-aging	[162]
	*Enteromorpha prolifera*	Immunomodulator, antioxidant, hypolipidemic	[139]
	*Ulva* sp.	Anti-adhesive, antiproliferative, hepatoprotective	[160]
	*U. pertusa*	Antioxidant, antiproliferative, hypocholesterolemic	[163]
	*U. pertusa*	Antioxidant, hypotriglyceridemic, decreases LDL- and increases HDL-cholesterol, immunostimulatory	[164]
Fucoidans	*Sargassum mcclurei*	Antiviral against HIV-1, blocks entry of the virus	[165]
	*Adenocytis utricularis* *Undaria pinnatifida* *Stoechospermum marginatum* *Cystoseira indica*	Antiviral against HSV-1, HSV-2, HCMV, VSV, Sindbis virus, and HIV-1	[166]
	*Ecklonia cava* *E. kurome*	Antiproliferative, antitumor, anticoagulant, antioxidant, antithrombotic, anti-inflammatory	[167,168]
	*S. horneri*	Antitumor, antiviral	[169]
	*Fucus* sp.	Immunostimulant, antiviral, antitumor, antiproliferative, antiadhesive	[148]
	*Ascophyllum nodosum*	Immunomodulatory, anti-inflammatory, anticoagulant, antithrombotic	[170]
	*Padina tetrastromatica*	Antitumor, antiviral	[171]
	*A. nodosum* *Cladosiphon okamuranus* *Fucus spiralis* *F. distichus*, *F. evanescens* *F. vesiculosus* *F. serratus* *L. digitata* *L. saccharina*	Anticoagulant, anti-inflammatory, antiadhesive, antiangiogenic	[170]
	*F. vesiculosus*	Anti-atopic dermatitis	[172]
	*F. evanescens*	Antitumor	[173]
	*S. fusiforme*	Antiangiogenic	[174]
	*S. fusiforme*	Anticancer	[175]
	*C. okamuranus*	Anticancer	[176]
	*Adenocystis utricularis*	Antiretroviral	[177]
	*A. utricularis*	Antiviral	[147]
	*C. okamuranus*	Cardioprotective	[178]
	*C. okamuranus*	Antiproliferative	[179]
	*C. okamuranus*	Gastric protection	[180]
	*C. okamuranus*	Antiprion	[181]
	*F. evanescens*	Anticoagulant	[182]
	*F. evanescens*	Anti-inflammatory	[183]
	*F. vesiculosus*	Anti-obesity	[184]
	*Undaria piaantifida*	Immunostimulatory	[185]
	*L. japonica*	Antioxidant	[186]
	*L. japonica*	Anti-inflammatory	[187]
	*Lessonia vadosa*	Anticoagulant and elicitor	[188]
	*U. pinnatifida*	Antiplasmodial	[189]
	*U. pinnatifida*	Anti-allergy	[190]
	*U. pinnatifida*	Antitumor	[191]
	*U. pinnatifida*	Antitumor	[192]
Laminarins	*Laminaria* sp.	Anti-inflammatory	[193]
	*Eisenia bicyclis*	Antibacterial	[194]
	*E. bicyclis*	Anticancer	[195]
	*L. digitata*	Antioxidant protection	[196]
	*L. japonica*	ROS scavenging potential	[197]
	*L. digitata*	Anticancer	[198]
Alginates	*L. hyperborea* *L. digitata* *L. japonica* *A. nodosum* *Macrocystis pyrifera*	Antiviral	[199]
	*Eucheuma cottonii* *S. polycystum*	Antidiabetic	[200]
	*Laminaria* sp.	Drug carriers	[201]
	*A. nodosum*	Scaffolds for ligaments and tissue engineering	[202]
	*Ecklonia* sp.	Regeneration of tissues	[203]
	*M. pyrifera*	Wound healing and dressing	[204]
	*L. hyperborean*	Wound healing	[205]

**Table 5:** Seaweed lipids and their biological properties.

Lipid Type	Algal Sources	Properties	References
Omega-3 fatty acids	Phaeophyta	Ant-inflammation, boosts brain function, supports eye health, improves heart health	[244]
Arachidonic acid	*Porphyridium cruentum*	Improves growth and development of neonates	[245]
Fucosterol	*Sargassum fusiformis*	Anti-aging	[246]
Docosahexaenoic acid	*Phaeophyta*	Cardiovascular health, eye and brain health, development of nervous system	[245]
Eicosapentaenoic acid	*Porphyridium cruentum*	Cognition, heart health, protection against arthrosclerosis, anti-inflammatory potential	[247]
Polyunsaturated fatty acid	*Undaria pinnatifida*	Anti-inflammation	[248]
Palmitic acid	*Fucus vesiculosus Saccharina latissima Gracilaria* sp. *Ulva rigida*	Enzyme inhibition, antioxidant	[249]
Fucosterol	*S. fusiformis* *Pelvetia siliquosa*	Anti-inflammatory, antioxidant, increased antioxidative enzymes	[250,251]
Essential oil	*Laminaria japonica* *Gracilaria verrucosa*	Anti-inflammatory, antioxidant, antibacterial activity	[252,253]
Phospholipids	*Grateloupia turuturu* *Ecklonia radiata* *Hormosira banksii*	Antioxidant and anti-inflammatory potential	[254,255]
Glycolipids	*Chondria armata* *Gracilaria corticata*	Antimicrobial activity	[256,257]

**Table 6:** The potential biological activity possessed by seaweed-derived bioactive compounds.

Biological Activity	Bioactive Compounds/Extracts	Seaweed Species	Reference
Antioxidant	Eckol	*Ecklonia cava* sub sp. *Stolonifera*	[296]
	Methanolic extract	*Osmundaria obtusiloba*	[297]
	Polysaccharide	*Mazzaella canaliculate*	[298]
	Fucoidan	*Sargassum fusiforme*	[299]
	Histidyl dipeptide	*Acanthophora nayadiformis*	[300]
	Aqueous extract	*Hypnea musciformis*	[301]
	Protein hydrolysates	*Gracilariopsis lemaneiformis*	[302]
	Crude phlorotannin	*Ecklonia stolonifera*	[303]
	Crude phlorotannin	*Eisenia bicyclis*	[303]
	Aqueous extract	*Laminaria ochroleuca*	[304]
	Polyphenol	*Ascophyllum nodosum*	[305]
	Polyphenol	*Pelvetia canaliculate*	[305]
	Polyphenol	*Fucus spiralis*	[305]
	Polyphenol	*Ulva intestinalis*	[305]
	Polyphenol	*Saccharina japonica*	[306]
Anti-allergic	Chlorophyll c2	*Sargassum horneri*	[307]
	Phlorotannin	*Fucus* sp.	[308]
	Methanolic extract	*Sargassum hemiphyllum*	[309]
	Methanolic extract	*Polyopes affinis*	[310]
	Aqueous extract	*Ecklonia cava*	[311]
	Seaweed powder	*Eisenia arborea*	[312]
Antidiabetic	Aqueous extract	*Halimeda macroloba*	[313]
	Oligosaccharide	*Sargassum confusum*	[314]
	Ethyl acetate extract	*Ulva lactuca*	[315]
	Polysaccharides	*Sargassum* sp.	[316]
	Aqueous extract	*Halymenia durvillei*	[317]
	Polysaccharide hydrolysates	*Sargassum confusum*	[318]
	Ethanolic extract	*Lessonia nigrescens*	[319]
Anticoagulant	Flavonoid	*Sargassum cristaefolium*	[320]
	Sulfated polysaccharide	*Gelidiella acerosa*	[321]
	Polysaccharide	*Sargassum fusiforme*	[322]
	Ulvans	*Ulva lactuca*	[323]
	Sulfated polysaccharide	*Ecklonia cava*	[324]
Antiviral	Sulfated polysaccharide	*Dictyota bartayesiana*	[325]
	Fucoidans	*Nizamuddinia zanardinii*	[326]
	Fucoidans	*Fucus distichus* subsp. *Evanescens*	[327]
	Phlorotannin	*Ecklonia cava*	[328]
	Crude extract	*Canistrocarpus cervicornis*	[329]
	Crude extract	*Osmundaria obtusiloba*	[330]
Antiproliferative	Polyphenol	*Ulva reticulata*	[331]
	Ethanolic extract	*Egregia menziesii*	[332]
	Fucoidan	*Nizamuddinia zanardinii*	[333]
	Phlorotannin	*Bifurcaria bifurcate*	[334]
	Fucoxanthin	*Phaeodactylum tricornutum*	[335]
	Sulfated polysaccharide	*Sargassum cinereum*	[336]
Immunomodulator	Sulfated polysaccharides	*Ulva prolifera*	[337]
	Fucoidans	*Lobophora variegate*	[338]
	b-1,3/1,6-glucan	*Durvillaea antarctica*	[339]
	Fucoidans	*Agarum clathratum*	[340]
	Enzymatic extract	*Ecklonia cava*	[341]
	Alginic acid	*Sargassum wightii*	[342]
Antihypertensive	Phlorotannins	*Ascophyllum nodosum*	[343]
	Chloroform: methanol extract	*Sargassum wightii*	[344]
	Aqueous extract	*Ulva linza*	[345]
	Protein content	*Macrocystis pyrifera*	[346]
	Fucopyranan	*Sargassum wightii*	[347]

## Data Availability

The data upon which this review was written were obtained from the referenced articles. The corresponding author agrees to be contacted if consultation is needed.

## Abbreviations

The following abbreviations are used in this manuscript:

FAO	Food and Agriculture Organization, United Nations
PUFA	Polyunsaturated fatty acids
ASFIS	Aquatic and fisheries information system
VEGF	Vascular endothelial growth factor
DHA	Docosahexaenoic
SOFIA	The State of World Fisheries and Aquaculture
FOSHU	Foods for specified health uses
